# Dangerous Industries

**Published:** 1899-07-15

**Authors:** 


### Dangerous Industries.

The debate originated by Sir Charles Dilke, in the House of Commons on Friday, on the subject of the regulation of dangerous trades, was so conducted as to show that the subject is not likely to be lost sight of by the Legislature, and that none of the ordinary official difficulties will be permitted to stand in the way of such reforms as may be practicable. The Home Secretary, although unable to give a definite pledge, yet expressed a strong hope that he might be able to introduce a Bill upon the subject at an early period of next session; and said that, in the meanwhile, by the more careful inspection which had been rendered possible by an increase of the staff, and by constant communications with employers, he was expecting to be able to mitigate the admitted evils in a considerable degree. The debate mainly turned upon the questions of lead poisoning in the Potteries and of poisoning by phosphorus in the match industry; but an alleged increase of anthrax among wool combers or wool sorters was also mentioned as a matter which called urgently for the attention of the Home Office. With regard to lead poisoning, the members of the House who had previously identified themselves with the question contended that the reports of Professors Thorpe and Oliver fully justified the position which they had assumed; but it seemed to be admitted on all hands that the substitution of "fritted" for ordinary lead in the glazing processes was likely to be attended by a considerable diminution, if not even by an entire disappearance, of the suffering and mortality which have hitherto been produced. This blessed word "fritted" was largely employed in the debate, although it may be doubted whether it conveyed, as far as the bulk of the members were concerned, any greater or more precise significance than that of "Mesopotamia" in the ears of the old lady who was a friend of our youth. As far as can be ascertained from Murray's Dictionary, it springs from the same root as "friture," and means that the substance to which it is applied has been exposed to a heat insufficient to cause fusion. In fact, the lead has been in some way "cooked"—a process from which, it may be hoped, the statements with regard to the advantages of using it have been exempt.

It has to be admitted that many things in the way of legislation are allowable for health's sake, even though they may smack of economic heresy. It may be doubted, and we know that by many it is seriously doubted, to what extent the legislature is justified in exercising parental control over the conduct of dangerous industries for the protection of adult males. It has been said many a time that with the enormous power possessed by trades unions and other combinations of workmen, a power the possession of which they not only admit but boast of, it ought to be the easiest thing in the world for working people to ensure for themselves good, healthy, and sanitary conditions of labour. And so it should be, but it is not done. They have the power, but seem lacking in the conviction of the necessity of using it for purposes of health; and whereas a whole shopful of "hands" will strike for a prospective halfpenny an hour, or in "sympathy" with another shop where a strike is going on, no notice is taken of the grossest errors in sanitation, and it is difficult at times even to induce workers to complain of such things to the inspectors. It is one of the saddest things, but unfortunately it is the fact, that in almost every endeavour which has been made for the amelioration of the health conditions of labour, it is the labourer himself that has been, and in many trades still is, the stumbling block, and it is because we are beginning to recognise as a nation that matters of health stand on a different footing from matters of wages that we have in many directions admitted the principle that health legislation of a parental character is permissible even though it may seem to interfere on the one hand with "personal liberty," and on the other with the "rights of capital." But while we admit that so precious is health to the community as well as to the individual, that health legislation may well be excused conforming in all points to economic laws, we cannot but see that such legislation is likely to be of but small avail if it is far in advance of the sanitary education of the people. It will be of small avail to enforce the employment of "fritted" lead in the glazing houses, if the women who are thus protected from one form of poisoning are found willing to subject themselves to another and more deadly one, by the practice of taking lead which has not been fritted, as a method of procuring abortion. It will be of small avail to render the surroundings of male labour more wholesome if the lives which may be thus prolonged are only prolonged in order to swell the percentages of mortality from the excessive use of alcohol. Sanitary legislation, if it is to be effectual for good, must always go hand-in-hand with sanitary education.

